# Associations of Meal Timing and Frequency with Obesity and Metabolic Syndrome among Korean Adults

**DOI:** 10.3390/nu11102437

**Published:** 2019-10-13

**Authors:** Kyungho Ha, YoonJu Song

**Affiliations:** Department of Food Science and Nutrition, The Catholic University of Korea, Gyeonggi 14662, Korea; kyungho.ha715@gmail.com

**Keywords:** nightly fasting duration, morning eating, night eating, meal frequency, obesity, metabolic syndrome, Korean

## Abstract

Emerging studies indicate that meal timing is linked to cardiometabolic risks by deterioration of circadian rhythms, however limited evidence is available in humans. This large-scale cross-sectional study explored the associations of meal timing and frequency with obesity and metabolic syndrome among Korean adults. Meal timing was defined as nightly fasting duration and morning, evening, and night eating, and meal frequency was estimated as the number of daily eating episodes using a single-day 24-hour dietary recall method. Meal frequency was inversely associated with prevalence of abdominal obesity, elevated blood pressure, and elevated triglycerides in men only. Independent of the nightly fasting duration and eating episodes, morning eating was associated with a lower prevalence of metabolic syndrome (odds ratio (OR), 0.73; 95% confidence interval (CI), 0.57–0.93 for men and OR, 0.69; 95% CI, 0.54–0.89 for women) than no morning eating, whereas night eating was associated with a 48% higher prevalence of metabolic syndrome (OR, 1.48; 95% CI, 1.15–1.90) than no night eating in men only. Longer fasting duration and less sleep were associated with obesity and metabolic syndrome. These findings suggest that overall eating patterns, including energy distribution across the day, eating frequency, and sleep duration, rather than fasting duration alone, are related to cardiometabolic risks in free-living Korean adults.

## 1. Introduction

Intermittent fasting has been a topic of interest in recent years. Compelling evidence in rodent models indicates that time-restricted feeding without reducing caloric intake protects against components of metabolic disease, such as obesity and hyperinsulinemia. The timing of food intake appears to affect the robustness of circadian rhythms in metabolic organs, and circadian rhythm disruption is emerging as a new risk factor for cardiovascular diseases [1,2,3].

The availability of artificial light in modern society has led to a nocturnal lifestyle [4] along with a longer period of food consumption, as the increased variety of methods to purchase foods has enabled easier and faster access to food than in the past [5,6]. In addition to these environmental changes, eating patterns have changed, including increased frequencies of skipping meals, snacking [7], and nighttime eating [8]. These eating patterns, including meal timing and frequency, have been proposed to influence weight control and cardiometabolic health in humans [6,8,9].

A delayed temporal meal distribution was reported to be associated with cardiometabolic risk factors, including obesity and metabolic syndrome [8,9]. The central clock is located in the suprachiasmatic nucleus of the hypothalamus and is entrained mainly by light, but there are similar clocks in peripheral tissues, such as the liver; the feeding signal is the dominant timing cue for these peripheral clocks [9,10].

Compared with rodent studies, there have been limited studies of the association between meal timing and cardiometabolic risks in humans. A recent controlled feeding trial demonstrated that early time-restricted feeding allowing eating for 6 h and restricted food intake after 15:00 for 5 weeks improved insulin sensitivity, blood pressure, and oxidative stress in the absence of weight loss among eight men with prediabetes [11]. Early time-restricted feeding also facilitated weight loss by reducing appetite and increased fat oxidation among 11 overweight adults [12]. Another crossover clinical trial in 29 men of normal-weight showed that restricting nighttime eating for more than 11 h for 2 weeks resulted in a 0.4 kg weight loss in the intervention group compared with a 0.6 kg weight gain in the control group [13]. Meal frequency has been used as an indirect indicator of the fasting period [10]. A randomized crossover clinical trial in 15 healthy adults of normal weight reported that body weight was reduced after consumption of one meal/day diet for 8 weeks, but no differences in the levels of serum lipids, glucose, of insulin were apparent, when subjects consumed one meal/day compared with three meals/day [14]. However, most clinical trials reported to date have been small-scale, short-term intervention studies.

Several observational studies were conducted in larger samples, but these studies were still limited. A study in 2650 adult women in the USA showed that a longer nightly fasting duration was associated with an 8% lower C-reactive protein level only among women who ate less than 30% of their total daily calories in the evening, while there was no association between eating frequency or fasting duration and the homeostatic model assessment of insulin resistance [15]. A study in 12,389 adults in Korea showed that a lower meal frequency was associated with an increased risk of metabolic syndrome in men only [16]. As meal timing is related to meal frequency and other dietary behaviors, such as late eating, more comprehensive evidence on the associations of meal timing and frequency with metabolic disease is needed.

As major risk factors for cardiovascular diseases, obesity and metabolic syndrome are critical worldwide issues. In Korea, 3 in 10 adults have obesity or the metabolic syndrome [17,18]. Korea has undergone rapid economic growth and unique nutrition transitions in the diet [19]. The digital infrastructure is highly developed, which has enabled Koreans to have a high ranking in internet usage worldwide [20], which in turn has led to extended screen time and reduced sleeping hours in Korea. Several studies reported that these factors are associated with health outcomes [21,22,23,24]. Therefore, meal timing and frequency may be important determinants of metabolic disease in Korea. This study was performed to explore meal timing and frequency using various variables, including nightly fasting duration and specific time periods such as morning and night, and to examine their associations with obesity and metabolic syndrome in Korean adults using national survey data.

## 2. Materials and Methods

### 2.1. Data and Study Participants

This study used data from the 2013–2017 Korea National Health and Nutrition Examination Survey (KNHANES), which is a continuous annual survey conducted by the Korea Centers for Disease Control and Prevention (KCDC). The KNHANES involves a health interview, health examination, and dietary survey conducted in a nationally representative sample of Koreans selected by a complex, multistage, probability sampling design. Details of the study procedures are available elsewhere [25]. The KNHANES was approved by the KCDC Institutional Review Board until 2014 and was conducted without deliberation, as this has been conducted by the state for the public weal since 2015. Written informed consent was obtained from all participants.

Among 27,220 initial participants aged 19 years or older who participated in 24-hour dietary recall, those with missing anthropometric or biochemical measurements (*n* = 3992), with a diagnosis of, or receiving medications for, diabetes, hypertension, or dyslipidemia (*n* = 8128), who were pregnant or lactating (*n* = 225), who reported an implausible energy intake (<500 kcal/day or >5000 kcal/day; *n* = 290), with insufficient meal time information (*n* = 2), and with insufficient information regarding sleep duration (*n* = 304) were excluded. Finally, a total of 14,279 participants (5854 men and 8425 women) were included in the analysis.

### 2.2. Meal Timing and Frequency

Dietary data were obtained using a single-day 24-hour dietary recall method. Under the administration of trained staff at the participants’ homes, participants reported all foods and beverages consumed within a 24-hour period before the survey, including the exact time when they were consumed. Meal frequency was estimated as the number of daily eating episodes defined as food or beverage intake of 1 kcal or more at a single time point [26]. Meal timing was evaluated using several variables, including nightly fasting duration and eating during specific time windows, such as morning, evening, and night. Nightly fasting duration was calculated by subtracting the hours elapsed between the first to last eating episodes for the dietary recall day from 24 h [27], and the first eating episode was regarded as having occurred from 05:00. Night eating was defined as eating after 21:00, and participants were categorized as non-night eaters, <25% of total energy consumed at night, and ≥25% of total energy consumed at night based on the definition of night eating syndrome [28]. Morning eating was defined as eating from 05:00 to 09:00 and evening eating from 18:00 to 21:00. Participants were categorized using the approximate value of the top quartile for percentage of energy consumed in the morning and evening from the total energy (25% and 40%, respectively). Thus, morning eating was divided into non-morning eaters, <25% of total energy consumed in the morning, and ≥25% of total energy consumed in the morning, and evening eating was divided into <40% and ≥40% of total energy consumed in the evening because most participants consumed foods or beverages in the evening.

### 2.3. Definitions of Obesity and Metabolic Syndrome

At a mobile examination center, anthropometric variables such as height, weight, and waist circumference, blood pressure, and biochemical variables were measured using calibrated equipment based on standardized procedures. Body mass index (BMI, kg/m^2^) was calculated by dividing body weight (kg) by the height squared (m^2^). Blood pressure was measured three times, and the mean value of the second and third readings was used. High-density lipoprotein (HDL)-cholesterol, triglyceride, and fasting glucose levels were measured in participants after fasting for at least 8 h.

Obesity was defined as BMI ≥ 25 kg/m^2^ according to the World Health Organization Asia-Pacific guidelines [29]. Based on the National Cholesterol Education Program Adult Treatment Panel III criteria [30] with the exception of waist circumference [31], metabolic syndrome was diagnosed in participants who had three or more of the following metabolic abnormalities: (1) Abdominal obesity (≥90 cm for men and ≥85 cm for women), (2) elevated blood pressure (systolic blood pressure ≥130 mmHg or diastolic blood pressure ≥85 mmHg), (3) reduced HDL-cholesterol (<40 mg/dL for men and <50 mg/dL for women), (4) elevated triglycerides (≥150 mg/dL), and (5) elevated fasting glucose (≥100 mg/dL).

### 2.4. Sociodemographic and Lifestyle Variables

Information on the sociodemographic and lifestyle variables of the study participants was obtained from health interviews. Education level was categorized as middle school or lower, high school, and college or higher, and household income level was categorized as lowest, lower middle, upper middle, and highest based on monthly household income quartiles. The type of work was classified as day worker, shift worker, and other. Physical activity was defined as “yes” if the participant performed vigorous intensity activity for at least 75 min, moderate intensity activity for at least 150 min, or an equivalent combination of moderate and vigorous intensity activities per week. Alcohol consumption was classified as “none” for those who never drank any type of alcoholic beverage or drank less than once a month during the past year, “moderate” for those who drank alcoholic beverages more than once a month during the past year, and “high” for those who drank more than seven glasses of alcoholic beverages for men or five glasses for women per occasion more than twice per week. Smoking status was grouped into “never” for those who had never smoked cigarettes or smoked <100 cigarettes in their lifetime, “former” for those who had smoked ≥100 cigarettes in their lifetime but were current non-smokers, and “current” for those who had smoked ≥100 cigarettes in their lifetime and were current smokers.

### 2.5. Statistical Analysis

All statistical analyses were performed using SAS 9.4 (SAS Institute, Cary, NC, US). The complex sampling design parameters of the KNHANES, including strata, cluster, and weight, were used in the PROC SURVEY procedure. All statistical tests were two-sided, and *p* < 0.05 was taken to indicate statistical significance.

All continuous variables are presented as means ± standard error of the mean and all categorical variables are presented as percentiles (%). Differences in general characteristics according to sex were examined by *t* test for continuous variables and the chi-square test for categorical variables. Multiple logistic regression analysis was performed to estimate odds ratios (ORs) and 95% confidence intervals (CIs) for obesity and metabolic syndrome and its components according to meal timing and frequency. Primary confounding variables were age, BMI (except for obesity and abdominal obesity), education, household income, type of work, survey period, alcohol consumption, smoking, physical activity, and total energy intake. Meal frequency was divided into quartiles of the number of daily eating episodes, and the lowest quartile was used as the reference. Nightly fasting duration was categorized into <8 h, 8–10 h, 10–12 h, 12–16 h, and ≥16 h. As 16:8 intermittent fasting is a popular time-restricted feeding regimen [32], nightly fasting for 16 h or more was used as the reference. Morning, evening, and night eating were evaluated as “yes” or “no” (not evening) and according to the energy intake during each time period. In the model of energy intake during each time period, the nightly fasting duration and number of daily eating episodes were additionally adjusted.

In addition, it was hypothesized that the nightly fasting duration may be differentially associated with obesity and metabolic syndrome according to sleeping status. Thus, the joint associations of nightly fasting duration and sleep duration with obesity and metabolic syndrome were examined. According to a previous study indicating that sleeping less or more than 7–8 h per night was associated with an increased prevalence of metabolic syndrome [33], sleep duration was divided into <6 h, 6–7 h, 7–8 h, and ≥8 h in the present study. Considering the distribution of nightly fasting duration by sleep duration, the nightly fasting durations were combined (<12 h and ≥12 h). In a multiple logistic regression analysis, participants who fasted for <12 h and slept for 7–8 h were regarded as the reference group, and the same confounders described above were controlled.

## 3. Results

### 3.1. General Characteristics of the Study Participants

The general characteristics of the study participants, including meal timing and frequency, are shown in Table 1. The mean age was 41.1 years in men and 41.7 years in women. There were significant differences in education, type of work, physical activity, alcohol consumption, and smoking between men and women (*p* < 0.0001 for all). The average number of daily eating episodes was 5.3 and was slightly higher in men than in women (*p* < 0.0001). The mean nightly fasting duration was 12.2 h and was longer in women than in men (*p* < 0.0001). Approximately half of the participants reported consuming any food or beverage in the morning (55.3%) or at night (48.4%), and 84.3% of participants consumed food or beverages in the evening. Women (11.7%) consumed slightly more energy than men (11.1%) in the morning (*p* = 0.0228), whereas men consumed more energy than women in the evening and at night (*p* < 0.0001 for all). Men consumed 12.1% of their energy after 21:00 and women consumed 7.7% of their energy after 21:00.

### 3.2. Associations of Meal Timing and Frequency with Obesity and Metabolic Syndrome

In the multiple logistic regression models, men in the highest quartile of the number of daily eating episodes (median 8 times/day) had lower prevalence of abdominal obesity (OR, 0.82; 95% CI, 0.69–0.98), elevated blood pressure (OR, 0.82; 95% CI, 0.68–0.99), and elevated triglycerides (OR, 0.81; 95% CI, 0.68–0.96) than those in the lowest quartile (median 4 times/day; Table 2). The number of daily eating episodes was not significantly associated with obesity or metabolic syndrome in women. Compared with fasting for more than 16 h/day, fasting 10–12 h/day was significantly associated with decreased prevalence of obesity (OR, 0.75; 95% CI, 0.57–0.98), abdominal obesity (OR, 0.70; 95% CI, 0.53–0.92), and elevated triglycerides (OR, 0.69; 95% CI, 0.53–0.91) in men, and significantly associated with a decreased prevalence of abdominal obesity (OR, 0.74; 95% CI, 0.56–0.98) in women. Morning eating was associated with decreased prevalence of metabolic syndrome (OR, 0.83; 95% CI, 0.70–0.99) and elevated triglycerides (OR, 0.76; 95% CI, 0.66–0.87) in men and with decreased prevalence of metabolic syndrome (OR, 0.71; 95% CI, 0.59–0.87), abdominal obesity (OR, 0.82; 95% CI, 0.70–0.95), and elevated fasting glucose (OR, 0.80; 95% CI, 0.69–0.92) in women. Night eating was significantly associated with increased prevalence of metabolic syndrome (OR, 1.25; 95% CI, 1.04–1.49) and reduced HDL-cholesterol (OR, 1.18; 95% CI, 1.01–1.38) in men only.

The significant associations of morning and night eating with metabolic syndrome and its components remained strong after additional adjustments for nightly fasting duration and number of daily eating episodes (Table 3). Among men and women who consumed 25% or more of their total energy in the morning, men showed decreased prevalence of metabolic syndrome (OR, 0.73; 0.95% CI, 0.57–0.93) and elevated triglycerides (OR, 0.73; 95% CI, 0.59–0.89), and women showed decreased prevalence of metabolic syndrome (OR, 0.69; 95% CI, 0.54–0.89) and elevated fasting glucose (OR, 0.65; 95% CI, 0.53–0.79), compared with non-morning eaters. Men who consumed 25% or more of their total energy at night showed a 48% increased prevalence of metabolic syndrome (OR, 1.48; 95% CI, 1.15–1.90) compared with non-night eaters. Evening eating was not associated with obesity or metabolic syndrome.

### 3.3. Joint Associations of Nightly Fasting Duration and Sleep Duration with Obesity and Metabolic Syndrome

**Figure 1:**
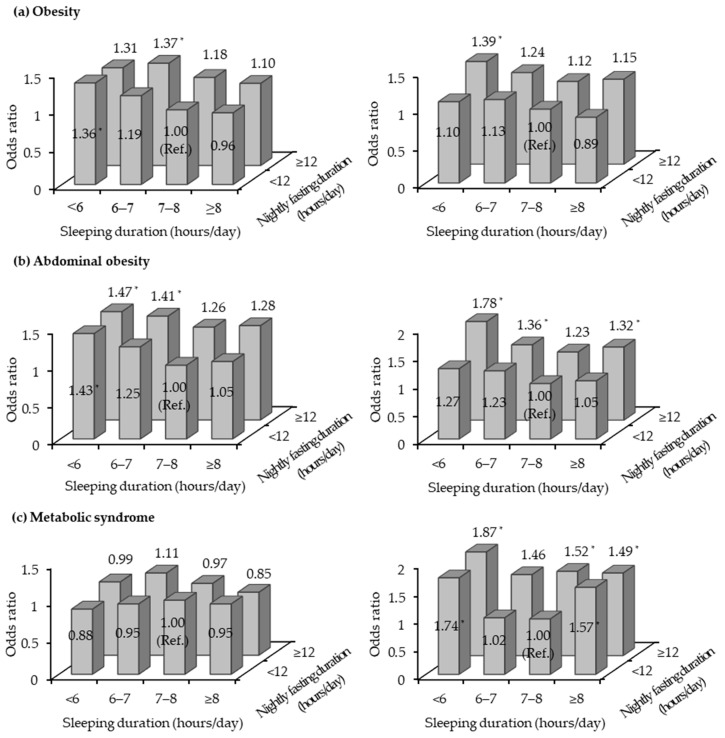
Multivariable-adjusted odds ratios for obesity (**a**), abdominal obesity (**b**), and metabolic syndrome (**c**) according to nightly fasting duration and sleep duration in men (left side) and women (right side). Adjusted for age, body mass index (except for obesity and abdominal obesity), education, household income, type of work, survey period, alcohol consumption, smoking, physical activity, and total energy intake. * indicates statistical significance.

Figure 1 shows the joint associations of nightly fasting duration and sleep duration with obesity and metabolic syndrome. Compared with women who fasted for <12 h and had a sleep duration of 7–8 h, those who fasted for ≥12 h and slept for <6 h showed the highest ORs for obesity (OR, 1.39; 95% CI, 1.08–1.79), abdominal obesity (OR, 1.78; 95% CI, 1.33–2.38), and metabolic syndrome (OR 1.87; 95% CI, 1.23–2.82). Similarly, the OR for abdominal obesity was highest among men who fasted for ≥12 h and slept for <6 h (OR, 1.47; 95% CI, 1.07–2.03).

## 4. Discussion

The results of the present study indicated that a greater number of eating episodes was associated with lower prevalence of metabolic abnormalities, including abdominal obesity, elevated blood pressure, and elevated triglycerides, in men. This was consistent with previous cross-sectional studies, which reported inverse associations of eating frequency with obesity among adults in the USA [34], obesity and abdominal obesity among Swedish men [35], and blood pressure among Korean adults [36]. However, there have been reports of the opposite finding, in that a lower eating frequency was associated with reduced risks of overweight or obesity and abdominal obesity among adults in the USA [37,38], and with lower levels of BMI and waist circumference in British adults [39].

These inconsistent findings may be explained by discrepancies in dietary assessments and definitions of eating frequency. Some studies, including the present study, assessed eating frequency based on 24-hour dietary recall data [34,37,38], whereas other studies were based on the participants’ responses using questionnaires [16,35,36]. In addition, some studies defined eating frequency based only on main meals [16,36], whereas eating frequency was defined as both meals and snacks in others, including the present study [34,35,37,38,39]. As snacking has become more prevalent and the timing of eating more variable, it is difficult to distinguish meals and snacks [7,9]. A recent study that monitored eating patterns using a smartphone application in healthy young adults showed that a breakfast/lunch/dinner temporal pattern was mostly absent, and participants in the top decile of eating frequencies ate caloric foods 10.6 times per day [40].

We did not find a significant linear association between nightly fasting duration and obesity or metabolic syndrome, although previous studies in rodent models [10] and humans [11,13] reported that a longer fasting duration had a protective effect against cardiometabolic risks. In this study, fasting for 10–12 h was associated with a reduced prevalence of abdominal obesity in both sexes and reduced prevalence of obesity and elevated triglycerides in men, compared with fasting for 16 h or longer.

Independent of the nightly fasting duration and number of eating episodes, morning and night eating were meaningful factors influencing metabolic syndrome in Korean adults. The prevalence of metabolic syndrome was inversely associated with morning eating in both men and women, whereas it was positively associated with night eating in men. Eating breakfast and night eating are associated with cardiometabolic risk factors. Previous studies indicated that regular daily breakfast eaters showed lower weight gain and decreased risks of reduced HDL-cholesterol, elevated blood pressure [41], and metabolic syndrome compared with those who eat breakfast infrequently [42]. On the other hand, night eating was associated with increased risks of obesity [43,44,45,46], dyslipidemia [45,47], hyperglycemia [48], and metabolic syndrome [44].

Other studies examined the associations of meal timing variables. A large-scale Japanese study reported that skipping breakfast with late-night dinner eating was associated with an increased risk of metabolic syndrome, but skipping breakfast alone showed no association with metabolic syndrome [44]. In a cross-sectional study of adult women in the USA, an inverse association between nightly fasting duration and C-reactive protein level was found only in women consuming <30% of total daily calories in the evening, whereas no such association was found in those consuming ≥30% of total daily calories in the evening [15]. These findings suggest that adverse metabolic outcomes may be attributable to several variables of meal timing as well as eating frequencies. Unlike animal studies and human clinical trials in well-controlled environments, participants in this study were in free-living, uncontrolled environments. Dashti et al. (2019) reported that a wide range of factors, such as physiological, behavioral, personal preferences, and cultural and environmental factors, influence meal timing [49]. Meal timing can be a risk factor, but other factors affect meal timing, and these should also be taken into consideration. Further comprehensive studies are necessary in free-living human subjects to examine these associations.

As sleeping is closely related to circadian rhythm, this study examined the association between the nightly fasting duration and sleep duration. The nightly fasting duration and sleep duration were modestly correlated, but a longer fasting duration and less sleep were associated with increased prevalence of obesity and metabolic syndrome. These findings are potentially explained in the context of the lifestyle of Korean adults. Korean adults, especially young adults, usually work longer, spend a great deal of time in front of smartphone, computer, and television screens, and sleep less [21,50,51]. Being awake until late at night can increase the tendency to eat at night and reduce the daily fasting duration, which are linked to increased risks of chronic diseases [10,49]. Our findings suggest that sleeping characteristics are factors that also contribute to meal timing.

In this study, eating frequency and night eating were significantly associated with cardiometabolic risk factors in men only. We could not fully explain why significant associations were found in men only. However, men had higher proportion of night eaters and greater energy intake at night than women that could be linked with other lifestyle factors such as high rate of drinking and smoking. These behavioral differences along with endocrine factors might affect cardiometabolic outcomes [52]. Monitoring eating patterns and lifestyle behaviors between men and women is necessary to elucidate the role of meal timing and frequency by sex in development and management of cardiovascular diseases.

Although meal timing and frequency in humans are affected by behavioral factors as well as physiological factors, its alternations can be used as one of the strategies to rebuild eating patterns under diverse environmental challenges such as longer night life and ease of access to food in modern society [6]. Given that risks of developing cardiovascular diseases increase after middle-aged adults, appropriate nutritional education or intervention programs regarding finding desirable eating patterns in early adult period are necessary.

The present study had some limitations. First, the cross-sectional design of the study makes it difficult to draw conclusions regarding the causal relationships of meal timing and frequency with obesity and metabolic syndrome. Second, the use of 1-day 24-hour dietary recall data may have been subject to recall bias of self-reported timing and measurement error due to within-subject variations. Although random measurement errors may attenuate the associations between dietary variables and health outcomes [53], we still found some significant associations of meal timing and frequency with cardiometabolic risk factors. Third, chronotype or psychological factors, which might affect eating patterns could not be examined due to the absence of related information, thus there might be potential confounding. Despite these limitations, to our knowledge, this is the first observational study to investigate the associations of meal timing and frequency with obesity and metabolic syndrome comprehensively, based on a nationally representative large sample of free-living Korean adults.

## 5. Conclusions

In conclusion, having desirable eating patterns, including eating in the morning and avoiding eating after 21:00, and an appropriate sleep schedule may be helpful for reducing the risks of obesity and metabolic syndrome, independently of fasting duration. Multilateral studies considering meal timing, frequency, and other behavioral factors by sex are needed to address the effects of meal timing on cardiometabolic health in free-living human subjects.

## Figures and Tables

**Table 1:** General characteristics of the study participants.

	Total (*n* = 14279)	Men (*n* = 5854)	Women (*n* = 8425)	*p* value
Age (years)	41.4 ± 0.2 ^1^	41.1 ± 0.2	41.7 ± 0.2	0.0106
Survey period				0.6674
2013–2015	8293 (58.4)	3397 (58.6)	4896 (58.3)	
2016–2017	5986 (41.6)	2457 (41.4)	3529 (41.7)	
Education				<0.0001
Middle school or lower	2953 (14.9)	1125 (12.7)	1828 (17.1)	
High school	5215 (39.6)	2161 (40.8)	3054 (38.4)	
College or higher	6061 (45.5)	2544 (46.5)	3517 (44.5)	
Household income				0.4495
Lowest	1737 (10.1)	733 (10.0)	1004 (10.3)	
Lower middle	3466 (23.7)	1413 (23.2)	2053 (24.1)	
Upper middle	4373 (31.8)	1808 (32.4)	2565 (31.3)	
Highest	4666 (34.4)	1885 (34.4)	2781 (34.3)	
Type of work				<0.0001
Day worker	8655 (62.7)	4092 (70.7)	4563 (54.7)	
Shift worker	1843 (14.7)	836 (16.2)	1007 (13.3)	
Other	3721 (22.5)	900 (13.1)	2821 (32.0)	
Physical activity ^2^				<0.0001
No	7339 (48.0)	2778 (44.1)	4561 (51.9)	
Yes	6874 (52.0)	3045 (55.9)	3829 (48.1)	
Alcohol consumption ^3^				<0.0001
None	6049 (37.5)	1600 (25.2)	4449 (49.8)	
Moderate	6698 (50.1)	3198 (55.9)	3500 (44.3)	
High	1521 (12.4)	1052 (18.9)	469 (5.9)	
Smoking ^4^				<0.0001
Never	9359 (61.0)	1716 (32.2)	7643 (90.0)	
Former	2288 (16.4)	1932 (28.2)	356 (4.5)	
Current	2619 (22.7)	2202 (39.6)	417 (5.6)	
Body mass index (kg/m^2^)	23.5 ± 0.0	24.2 ± 0.1	22.7 ± 0.0	<0.0001
Waist circumference (cm)	80.1 ± 0.1	84.4 ± 0.1	75.9 ± 0.1	<0.0001
Systolic blood pressure (mmHg)	113.8 ± 0.2	117.4 ± 0.2	110.1 ± 0.2	<0.0001
Diastolic blood pressure (mmHg)	75.0 ± 0.1	77.8 ± 0.2	72.1 ± 0.1	<0.0001
HDL-cholesterol (mg/dL)	52.0 ± 0.1	48.1 ± 0.2	56.0 ± 0.2	<0.0001
Triglycerides (mg/dL)	127.0 ± 1.1	153.5 ± 1.9	100.3 ± 0.9	<0.0001
Fasting blood glucose (mg/dL)	94.7 ± 0.2	96.5 ± 0.3	92.8 ± 0.2	<0.0001
Sleep duration (hours/day)	6.9 ± 0.0	6.9 ± 0.0	6.9 ± 0.0	0.0904
Total energy intake (kcal/day)	2087.7 ± 9.3	2403.0 ± 13.7	1769.6 ± 8.8	<0.0001
Eating episodes (times/day)	5.3 ± 0.0	5.5 ± 0.0	5.2 ± 0.0	<0.0001
Eating episodes (times/day) (median (Q1–Q3))	4.6 (3.5–5.9)	4.7 (3.5–6.1)	4.6 (3.6–5.7)	
Nightly fasting duration (hours/day)	12.2 ± 0.0	11.9 ± 0.0	12.5 ± 0.0	<0.0001
Nightly fasting duration (hours/day) (median (Q1–Q3))	12.0 (10.5–13.9)	12.0 (10.0–13.4)	12.4 (11.0–14.0)	
Morning eating (05:00–09:00) (yes)	8529 (55.3)	3678 (56.8)	4851 (53.7)	0.0011
Morning energy (kcal/day)	233.3 ± 3.3	262.7 ± 5.0	203.7 ± 3.5	<0.0001
Morning energy (% of total energy)	11.4 ± 0.2	11.1 ± 0.2	11.7 ± 0.2	0.0228
Evening eating (18:00–21:00) (yes)	12158 (84.3)	4984 (84.2)	7174 (84.4)	0.7587
Evening energy (kcal/day)	614.1 ± 5.8	726.8 ± 9.5	500.4 ± 5.5	<0.0001
Evening energy (% of total energy)	29.0 ± 0.2	30.0 ± 0.3	28.0 ± 0.3	<0.0001
Night eating (after 21:00) (yes)	6273 (48.4)	2921 (54.5)	3352 (42.3)	<0.0001
Night energy (kcal/day)	227.5 ± 4.9	311.6 ± 8.3	142.7 ± 3.7	<0.0001
Night energy (% of total energy)	9.9 ± 0.2	12.1 ± 0.3	7.7 ± 0.2	<0.0001

^1^ All values are presented as means ± SE or *n* (%) and the complex sampling design parameters of the KNHANES, including strata, cluster, and weight, were used in the PROC SURVEY procedure. ^2^ Physical activity: “yes”, performed vigorous intensity activity for at least 75 min, moderate intensity activity for at least 150 min, or an equivalent combination of moderate and vigorous intensity activities per week. ^3^ Alcohol consumption: “none”, no consumption of any type of alcoholic beverage or consumed alcoholic beverages less than once per month during the past year, “moderate”, consumed alcoholic beverages more than once per month during the past year, “high”, consumed more than seven glasses of alcoholic beverages for men or five glasses for women per occasion more than twice per week. ^4^ Smoking: “never”, never smoked cigarettes or smoked <100 cigarettes over a lifetime, “former”, smoked ≥100 cigarettes over a lifetime but not a current smoker, “current”, smoked ≥100 cigarettes over a lifetime and a current smoker.

**Table 2:** Multivariable-adjusted odds ratios and 95% confidence intervals for obesity and metabolic syndrome according to meal timing and frequency.

**Men (*n* = 5854)**	**Obesity**	**Metabolic Syndrome**	**Abdominal Obesity**	**Elevated Blood Pressure**	**Reduced HDL-Cholesterol**	**Elevated Triglycerides**	**Elevated Fasting Glucose**
Eating episodes (times/day)							
Q1 (median 4) (*n* = 1968)	1.00 ^1,2^	1.00	1.00	1.00	1.00	1.00	1.00
Q2 (median 5) (*n* = 1213)	1.02 (0.86–1.20)	0.93 (0.74–1.18)	0.95 (0.79–1.15)	0.95 (0.79–1.14)	0.90 (0.72–1.11)	0.93 (0.77–1.12)	1.10 (0.91–1.32)
Q3 (median 6) (*n* = 1062)	0.85 (0.70–1.04)	0.80 (0.62–1.03)	0.86 (0.69–1.06)	0.85 (0.70–1.02)	0.92 (0.73–1.16)	0.79 (0.65–0.96)	0.88 (0.71–1.08)
Q4 (median 8) (*n* = 1611)	0.95 (0.81–1.12)	0.84 (0.67–1.05)	0.82 (0.69–0.98)	0.82 (0.68–0.99)	0.93 (0.76–1.14)	0.81 (0.68–0.96)	1.08 (0.90–1.30)
Nightly fasting duration (hours/day)							
<8 (*n* = 300)	0.83 (0.59–1.17)	0.97 (0.60–1.59)	0.78 (0.54–1.13)	0.82 (0.55–1.21)	0.84 (0.54–1.29)	0.71 (0.50–1.02)	1.17 (0.77–1.78)
8–10 (*n* = 897)	0.82 (0.62–1.09)	1.02 (0.69–1.50)	0.74 (0.54–1.01)	0.90 (0.66–1.24)	0.99 (0.69–1.41)	0.80 (0.60–1.08)	1.32 (0.95–1.85)
10–12 (*n* = 1756)	0.75 (0.57–0.98)	0.83 (0.58–1.19)	0.70 (0.53–0.92)	0.83 (0.63–1.10)	0.84 (0.60–1.18)	0.69 (0.53–0.91)	1.25 (0.91–1.71)
12–16 (*n* = 2463)	0.85 (0.66–1.09)	0.91 (0.63–1.30)	0.81 (0.62–1.06)	0.94 (0.72–1.23)	0.89 (0.65–1.23)	0.83 (0.64–1.08)	1.22 (0.89–1.68)
≥16 (*n* = 438)	1.00	1.00	1.00	1.00	1.00	1.00	1.00
Morning eating							
No (*n* = 2176)	1.00	1.00	1.00	1.00	1.00	1.00	1.00
Yes (*n* = 3678)	0.95 (0.83–1.09)	0.83 (0.70–0.99)	0.87 (0.75–1.00)	1.00 (0.86–1.16)	0.87 (0.73–1.03)	0.76 (0.66–0.87)	0.99 (0.86–1.15)
Night eating							
No (*n* = 2933)	1.00	1.00	1.00	1.00	1.00	1.00	1.00
Yes (*n* = 2921)	0.89 (0.78–1.01)	1.25 (1.04–1.49)	0.89 (0.78–1.02)	0.95 (0.83–1.10)	1.18 (1.01–1.38)	1.06 (0.92–1.22)	1.08 (0.94–1.25)
**Women (*n* = 8425)**	**Obesity**	**Metabolic Syndrome**	**Abdominal Obesity**	**Elevated Blood Pressure**	**Reduced HDL-Cholesterol**	**Elevated Triglycerides**	**Elevated Fasting Glucose**
Eating episodes (times/day)							
Q1 (median 4) (*n* = 2789)	1.00	1.00	1.00	1.00	1.00	1.00	1.00
Q2 (median 5) (*n* = 2171)	0.86 (0.73–1.00)	1.04 (0.82–1.32)	0.92 (0.77–1.10)	0.93 (0.77–1.14)	0.96 (0.83–1.10)	0.88 (0.72–1.08)	1.09 (0.91–1.31)
Q3 (median 6) (*n* = 1756)	0.93 (0.78–1.12)	0.92 (0.71–1.20)	0.95 (0.77–1.16)	0.90 (0.73–1.12)	1.07 (0.91–1.24)	0.88 (0.71–1.08)	1.01 (0.83–1.23)
Q4 (median 7) (*n* = 1709)	0.90 (0.76–1.07)	0.95 (0.73–1.25)	0.88 (0.72–1.08)	1.01 (0.83–1.24)	1.09 (0.92–1.28)	0.90 (0.74–1.11)	1.03 (0.84–1.26)
Nightly fasting duration (hours/day)							
<8 (*n* = 180)	0.76 (0.48–1.21)	0.59 (0.29–1.19)	0.63 (0.36–1.09)	0.61 (0.34–1.11)	1.13 (0.75–1.71)	1.20 (0.72–1.99)	0.95 (0.57–1.61)
8–10 (*n* = 783)	0.87 (0.65–1.16)	0.71 (0.45–1.11)	0.80 (0.56–1.13)	1.02 (0.69–1.51)	0.90 (0.69–1.18)	0.82 (0.57–1.18)	1.00 (0.72–1.38)
10–12 (*n* = 2290)	0.79 (0.62–1.00)	0.76 (0.52–1.12)	0.74 (0.56–0.98)	0.92 (0.65–1.29)	0.96 (0.77–1.20)	0.93 (0.68–1.28)	0.96 (0.73–1.26)
12–16 (*n* = 4451)	0.92 (0.74–1.16)	0.91 (0.64–1.29)	0.90 (0.70–1.16)	0.98 (0.71–1.35)	1.08 (0.88–1.32)	0.95 (0.71–1.27)	0.96 (0.74–1.24)
≥16 (*n* = 721)	1.00	1.00	1.00	1.00	1.00	1.00	1.00
Morning eating							
No (*n* = 3574)	1.00	1.00	1.00	1.00	1.00	1.00	1.00
Yes (*n* = 4851)	0.91 (0.80–1.03)	0.71 (0.59–0.87)	0.82 (0.70–0.95)	0.94 (0.80–1.11)	0.90 (0.80–1.01)	0.89 (0.76–1.03)	0.80 (0.69–0.92)
Night eating							
No (*n* = 5073)	1.00	1.00	1.00	1.00	1.00	1.00	1.00
Yes (*n* = 3352)	0.91 (0.81–1.03)	1.00 (0.82–1.21)	0.96 (0.83–1.11)	0.95 (0.81–1.12)	1.02 (0.91–1.15)	1.05 (0.91–1.22)	1.07 (0.93–1.24)

^1^ The complex sampling design parameters of the KNHANES, including strata, cluster, and weight, were used in the PROC SURVEY procedure. ^2^ Adjusted for age, body mass index (except for obesity and abdominal obesity), education, household income, type of work, survey period, alcohol consumption, smoking, physical activity, and total energy intake.

**Table 3:** Multivariable-adjusted odds ratios and 95% confidence intervals for obesity and metabolic syndrome according to energy intake in the morning, evening, and night.

**Men (*n* = 5854)**	**Obesity**	**Metabolic Syndrome**	**Abdominal Obesity**	**Elevated Blood Pressure**	**Reduced HDL-Cholesterol**	**Elevated Triglycerides**	**Elevated Fasting Glucose**
Morning energy (% of total energy)							
None (*n* = 2176)	1.00 ^1,2^	1.00	1.00	1.00	1.00	1.00	1.00
<25% (*n* = 2293)	0.97 (0.83–1.14)	0.85 (0.69–1.04)	0.91 (0.77–1.09)	1.10 (0.92–1.31)	0.83 (0.68–1.01)	0.78 (0.66–0.93)	1.01 (0.85–1.20)
≥25% (*n* = 1385)	1.07 (0.89–1.30)	0.73 (0.57–0.93)	0.99 (0.81–1.21)	0.92 (0.75–1.13)	0.89 (0.71–1.11)	0.73 (0.59–0.89)	0.86 (0.70–1.04)
Night energy (% of total energy)							
None (*n* = 2933)	1.00	1.00	1.00	1.00	1.00	1.00	1.00
<25% (*n* = 1918)	0.96 (0.82–1.13)	1.30 (1.04–1.63)	1.00 (0.85–1.19)	0.96 (0.81–1.15)	1.25 (1.04–1.51)	1.17 (0.98–1.39)	1.07 (0.90–1.27)
≥25% (*n* = 1003)	0.86 (0.71–1.04)	1.48 (1.15–1.90)	0.92 (0.75–1.15)	1.00 (0.81–1.23)	1.26 (1.00–1.60)	1.22 (0.99–1.49)	1.06 (0.85–1.33)
Evening energy (% of total energy)							
<40% (*n* = 4153)	1.00	1.00	1.00	1.00	1.00	1.00	1.00
≥40% (*n* = 1701)	1.07 (0.94–1.22)	1.03 (0.86–1.25)	0.96 (0.83–1.12)	1.08 (0.93–1.25)	1.05 (0.89–1.23)	0.94 (0.81–1.09)	1.12 (0.96–1.29)
**Women (*n* = 8425)**	**Obesity**	**Metabolic Syndrome**	**Abdominal Obesity**	**Elevated Blood Pressure**	**Reduced HDL-Cholesterol**	**Elevated Triglycerides**	**Elevated Fasting Glucose**
Morning energy (% of total energy)							
None (*n* = 3574)	1.00	1.00	1.00	1.00	1.00	1.00	1.00
<25% (*n* = 2838)	0.93 (0.79–1.10)	0.79 (0.62–1.01)	0.87 (0.72–1.04)	1.00 (0.82–1.21)	0.93 (0.81–1.06)	0.93 (0.77–1.12)	0.83 (0.70–0.99)
≥25% (*n* = 2013)	1.00 (0.84–1.19)	0.69 (0.54–0.89)	0.85 (0.69–1.04)	0.93 (0.76–1.14)	0.90 (0.77–1.06)	0.85 (0.70–1.04)	0.65 (0.53–0.79)
Night energy (% of total energy)							
None (*n* = 5073)	1.00	1.00	1.00	1.00	1.00	1.00	1.00
<25% (*n* = 2468)	0.96 (0.82–1.13)	1.19 (0.93–1.53)	1.12 (0.94–1.34)	0.98 (0.80–1.20)	1.08 (0.94–1.25)	1.16 (0.96–1.39)	1.08 (0.90–1.30)
≥25% (*n* = 884)	1.00 (0.81–1.24)	1.10 (0.77–1.58)	0.92 (0.71–1.20)	0.98 (0.74–1.29)	1.10 (0.90–1.34)	1.07 (0.82–1.38)	1.14 (0.89–1.45)
Evening energy (% of total energy)							
<40% (*n* = 6273)	1.00	1.00	1.00	1.00	1.00	1.00	1.00
≥40% (*n* = 2152)	1.14 (0.99–1.31)	0.99 (0.79–1.25)	1.10 (0.93–1.30)	1.20 (1.00–1.43)	1.08 (0.95–1.23)	1.04 (0.87–1.25)	0.98 (0.83–1.15)

^1^ The complex sampling design parameters of the KNHANES, including strata, cluster, and weight, were used in the PROC SURVEY procedure. ^2^ Adjusted for age, body mass index (except for obesity and abdominal obesity), education, household income, type of work, survey period, alcohol consumption, smoking, physical activity, total energy intake, nightly fasting duration, and number of daily eating episodes.
